# The Acute Effect of Upper-Body Complex Training on Power Output of Martial Art Athletes as Measured by the Bench Press Throw Exercise

**DOI:** 10.2478/hukin-2013-0079

**Published:** 2013-12-31

**Authors:** Loudovikos Dimitrios Liossis, Jacky Forsyth, Ceorge Liossis, Charilaos Tsolakis

**Affiliations:** 1 Department of Athletics, Deree, The American College of Greece.; 2 Department of Health, Staffordshire University.; 3 Department of Economics, Charagionis S.A.; 4 Department of Physical Education and Sports Sciences, Athens University.

**Keywords:** conditioning, Myotest, potentiation, power

## Abstract

The purpose of this study was to examine the acute effect of upper body complex training on power output, as well as to determine the requisite preload intensity and intra-complex recovery interval needed to induce power output increases. Nine amateur-level combat/martial art athletes completed four distinct experimental protocols, which consisted of 5 bench press repetitions at either: 65% of one-repetition maximum (1RM) with a 4 min rest interval; 65% of 1RM with an 8 min rest; 85% of 1RM with a 4 min rest; or 85% of 1RM with an 8 min rest interval, performed on different days. Before (pre-conditioning) and after (post-conditioning) each experimental protocol, three bench press throws at 30% of 1RM were performed. Significant differences in power output pre-post conditioning were observed across all experimental protocols (F=26.489, partial eta2=0.768, p=0.001). Mean power output significantly increased when the preload stimulus of 65% 1RM was matched with 4 min of rest (p=0.001), and when the 85% 1RM preload stimulus was matched with 8 min of rest (p=0.001). Moreover, a statistically significant difference in power output was observed between the four conditioning protocols (F= 21.101, partial eta^2^=0.913, p=0.001). It was concluded that, in complex training, matching a heavy preload stimulus with a longer rest interval, and a lighter preload stimulus with a shorter rest interval is important for athletes wishing to increase their power production before training or competition.

## Introduction

Complex training incorporates the execution of a resistance exercise before a biomechanically similar plyometric exercise during the same training session (Comyns et al., 2006; Matthews and Comfort, 2008; Robins et al., 2009). Several researchers have demonstrated the positive effects of complex training on the acute enhancement of both upper and lower body power (Baker, 2003; Matthews and Comfort, 2008); nevertheless, the results of complex-training studies have been equivocal given the number of factors that need to be taken into consideration, such as an intra-complex rest interval and adequate resistive load for the initial potentiating exercise, among others (Docherty and Hodgson, 2007; Ebben, 2002; Farup and Sorensen, 2010; Robbins, 2005).

Upper-body complex training has not received substantial attention compared with lower body complex training (Ebben, 2002). Moreover, results for the upper body seem more equivocal and less favourable than results for the lower body (Baker, 2003; Ebben, 2002; Farup and Sorensen, 2010; McGregor, 2006). In acute upper body complex training research studies, it has been demonstrated that 5–6 repetitions (reps) of heavy load strength exercises (i.e. bench press, bench pulls) at 65% to 85% of one repetition-maximum (1RM) alternated with 3 reps of light load plyometric exercises at 30% to 45% of 1RM (i.e. bench press throws, medicine ball putt) can induce significant improvements in power output as measured by the use of rotary/linear encoders/transducers (Baker, 2003; Baker and Newton, 2005; Ebben et al., 2000; Evans et al., 2000). However, in other acute training studies, researchers did not detect any effect of 5 reps of a heavy load strength exercise (bench press), at varying intensities of 1RM (87.5%, 66%, 44%), on power as measured by either 3 reps of an explosive power exercise at 45% of 1RM (bench throws) (Brandenburg, 2005) or 3 maximal effort explosive push-ups (Hrysomallis and Kidgell, 2001). Such discrepancies mainly stem from the fact that the optimal resistive load for the strength exercise and the rest interval between the strength and power exercise have not been clearly established (Robbins, 2005).

The physiological rationale for complex training is post-activation potentiation (PAP), which results in an improvement of the explosive capacity of the muscle stimulated by either maximal or sub-maximal prior contractile activity (Docherty et al., 2004). However, potentiation coexists with fatigue and the force a muscle is able to produce after previous contractile activity is the result of the net balance of fatigue and potentiation (Docherty and Hodgson, 2007). Moreover, the recovery interval and pre-load intensity load seem to be crucial additional factors that can influence PAP effect (Chiu et al., 2003).

The manifestation of PAP through the application of upper-body complex training has incited research interest although additional acute studies must confirm the positive effect of complex training on the acute enhancement of upper body power and to establish the appropriate variables (pre-load intensity, and recovery intervals between the strength and the power exercise) needed to induce power output increases. Thus, in order to provide effective guidelines concerning the use of complex training, the purpose of this study was to examine the acute effect of upper-body complex training on bench press throw power output (Baker, 2003), as well as to investigate the combined effect of two different resistive loads (65% 1RM versus 85% 1RM) for the initial potentiating bench press exercise (Matthews and Comfort, 2008) and two different intra-complex rest intervals (4 versus 8 min) between the strength and power exercise (Comyns et al., 2006). It was hypothesized that complex training could yield a significant change in power output production. It was further hypothesized that there would be significant differences in power output as a result of changes in the conditioning protocols (65% 1RM versus 85% 1RM pre-load, followed by a 4 versus 8 min rest interval). Both heavier and lighter loads are thought to activate PAP (Smilios et al., 2005), following rest within the suggested time frame intervals (Ferreira et al., 2012).

## Material and Methods

### Participants

Nine amateur-level combat sports/martial art athletes (boxing n = 2, kick-boxing n = 2, taekwondo n = 3 and karate n = 2) voluntarily participated in this study. The participants’ characteristics are presented in Table 1. All of these athletes had similar competition neuromuscular characteristics and had been undertaking a resistance training program for at least 6 months preceding the commencement of this study. Eligible participants did not report any subjective evidence of musculoskeletal disorders, and demonstrated a 1RM bench press equal to or in excess of their body mass (following the protocol of Farup and Sorensen (2010)). None of the participants had prior experience with the performance of plyometric bench press throws. Ethical approval was granted by the Department of Physical Education and Sports Science, University of Athens research ethics committee and prior to participating in this experiment, participants signed an informed consent form and a modified physical activity readiness questionnaire (medical screening). All procedures of this study were in accordance with the Helsinki declaration of 1975, as revised in 1996.

### Measures

Power output during each bench press throw was measured by the use of a three-dimensional accelerometer (Myotest Pro, Myotest S.A., France), which is a wireless handheld device, designed to provide a quantitative assessment of various measures of power performance. The Myostest equipment has demonstrated high reliability (R^2^ = 0.93 (linear regression), 0.955<r<0.994 for maximum power, r=0.94 for average power) as well as construct-validity in testing and measuring power and strength in men and women of various strength and fitness ability (Casartelli et al., 2010).

### Procedures

The nine participants involved in this study were required to visit the American College of Greece (ACG) Fitness-Wellness Institute for testing on five separate occasions. The primary objective of the first testing occasion was to determine the participants’ bench press 1RM (Bevan et al., 2009), according to the protocol of Kraemer and Fry (1995), so as to establish the relative loads for the bench press and the Smith-machine bench throw exercises, which formed the basis of the complex training protocols. In this session participants were also familiarized with the bench press throw exercise in order to minimize a potential learning effect (Thomas et al., 2007). After this initial visit, testing occurred on non-consecutive days, during which participants performed one of the four conditioning protocols in random order. All testing was conducted at the same time of the day (late morning) to eliminate a potential time-of-day effect (Farup and Sorensen, 2010; Martin et al., 1999). Participants were asked to abstain from any strenuous upper body exercise the day preceding testing.

At the beginning of each experimental session the participants completed a warm-up protocol lasting 5 min and consisting of low intensity jogging followed by 4 sets of sub-maximal bench presses (30%–50% 1RM) with 1.5 min rest between sets to prepare them for the subsequent intervention. This warm-up was similar to that carried out by Farup and Sorensen (2010) with the only difference that the cycle ergometer was substituted for a treadmill. The warm-up was not accompanied by stretching so as to limit any potential inhibition of power output production in the subsequent bench press throws (Thomas et al., 2007). The experimental conditions were timed as follows: Preconditioning (3 bench press throws at 30% 1RM), 60 s rest, conditioning protocol (either: 5 bench presses at 65% 1RM followed by 4 min of rest (65%–4); 5 bench presses at 65% 1RM followed by 8 min of rest (65%–8); 5 bench presses at 85% 1RM followed by 4 min rest (85%–4); or 5 bench presses at 85% 1RM followed by 8 min of rest (85%–8), post-conditioning (3 bench press throws at 30% of 1RM). All participants completed all experimental conditions. The preload stimulus of 65% of 1RM with a 3 min intra-complex rest interval and the preload stimulus of 87% of 1RM with an 8 min rest interval were shown to generate power output increases as constituents of complex training protocols; hence, they influenced the selection of this study’s variables (Baker, 2003; Bevan et al., 2009). Three bench press throws at 30% 1RM can induce power output maximization; hence, they were deemed adequate for this study (Falvo et al., 2006; Thomas et al., 2007).

#### Strength Testing (1RM)

Strength testing (1RM) was performed on a Cybex Bench Press by the use of an Eleiko barbell, following a standardised warm-up. Before the start of the strength testing session all participants underwent a warm-up protocol consisting of 5 min light intensity jogging on the treadmill followed by some static and dynamic stretching exercises (the emphasis was placed upon the chest and shoulder musculature that are heavily associated with the bench press exercise) (Bevan et al., 2009). A warm-up set of 5–10 reps was executed using 40%–60% of the perceived maximum 1RM. After a 1 min rest period, a set of 2–3 reps was performed at 60–80% of the perceived 1RM. The participants were expected to reach their 1RM effort within 3–5 maximal attempts (Thomas et al., 2007). Participants were instructed to grip the barbell slightly wider than shoulder width apart and lower the barbell under control till it nearly touched the chest; the participants then pushed the barbell back to a straight-arm (almost elbow lockout) position while keeping their feet and hips in contact with the foot stands and bench respectively (Baker, 2003). Participants were asked to execute each bench press repetition at a specific tempo (approximately 1.5 s for the concentric and 1.5 s for the eccentric phase) and use full range of motion (Brandenburg, 2005). Spotters were present to verbally encourage the participants and ensure their safety.

#### Concentric-Eccentric Bench Press Throws (Smith-Machine)

For the measurement of upper body power, participants completed three bench press throws on a Smith-machine 60 s before (pre-conditioning) and 4 or 8 min after (post-conditioning) the conditioning protocols with a relative load of 30% of 1RM. Successful upper body complex training protocols required participants to perform three bench press throw repetitions at 30–45% of 1RM (power exercise) since this resistance is purported to yield the maximum power output (Baker and Newton, 2005; Farup and Sorensen, 2010). For the bench press throws the participants took the same lifting position as with the bench press, with feet placed on the floor, and utilized the same grip that was employed in 1RM bench press testing; then the subject was instructed to start the movement by throwing the barbell as high as possible at the end of the concentric movement (Alemany et al., 2005). The subject then caught the barbell on its descent (eccentric movement) and instantaneously, without rest performed another maximal bench throw (concentric movement) (Alemany et al., 2005). Participants completed this plyometric sequence for 3 reps, with the aim of activating the stretch-shortening cycle (Alemany et al., 2005; Villarreal et al., 2010). Although it is well known that free weights are following an S or reverse C patterns path which may also activate secondary muscles and develop the ability to react under unstable conditions producing more force and power, we decided to use a Smith machine because it is commonly used when assessing bench press power performance, since the vertical lifting bar slides along a track, allowing the participant to lift heavy weights without any assistance, increasing at the same time the safety conditions (Schick et al., 2010; Vingren et al., 2011)

### Statistical Analysis

A two (time: pre-conditioning and postconditioning) × 4 (conditioning protocols) analysis of variance (ANOVA) with repeated measures was performed to determine differences in maximum power output between pre- and post-conditioning and between the four conditioning protocols. A one-way ANOVA with repeated measures (the four experimental conditions) was also performed by using the differences between post- and pre conditioning power output data. In cases where significant F values were identified (p<0.05), paired comparisons were employed in combination with the Holm’s Bonferroni method in order to control type I error and detect the exact location of these differences. Based on a power analysis, which was conducted before the commencement of this experiment, nine participants were needed to establish a statistical power of 0.80. The data used to conduct the power analysis were the Smith-machine barbell displacement data (Pre-Trial: 35.3(1.4) cm, Post-Trial: 37.2(1.4) cm) taken from the study of Bevan et al. (2009).

## Results

The pre- and post-conditioning power and standard deviation values for each conditioning protocol are presented in Table 2. All four conditioning protocols yielded a significant change in maximum power output between preand post conditioning values. Thus, there was a significant effect for time (Wilk’s Lambda=0.232, F (1,8)=26.5, p=0.001, multivariate partial eta^2^=0.768, power=0.994). However, there was no significant difference in power output between the four different conditioning protocols (Wilk’s Lambda=0.825, F (3,6)=0.423, p=0.743, multivariate partial eta^2^=0.175, power=0.096). Moreover, there was a significant interaction effect between pre- post-conditioning power output data and the four distinct conditioning protocols (Wilk’s Lambda=0.909, F (3,6)=19.96, p=0.002, multivariate partial eta^2^=0.909, power=0.997). Post-hoc analyses and pairwise comparisons revealed the exact location of significant differences between preand post-conditioning power output; 65%–4 induced significant power output increases (p = 0.001), 65%–8 induced mean power output decreases that were not significant due to the severe Bonferronni accepted p level (p = 0.033), 85%–4 induced significant power output decreases (p = 0.025), and 85%–8 induced significant power output increases (p = 0.001), respectively.

Using the one-way, repeated measures ANOVA, a significant difference for mean change (post-conditioning minus pre-conditioning) in maximum power output between the four conditioning protocols (according to the multivariate tests’ table) was observed. Thus, there was a significant effect for the conditioning protocol (Wilk’s Lambda=0.087, F (3, 6) = 21.101, p=0.001, multivariate partial eta^2^=0.913, power=0.998). The Mauchly’s test of sphericity was satisfied (p>0.05). Post-hoc analyses and pairwise comparisons revealed that there was a significant difference between: Conditioning protocol 1 (65%–4) and conditioning protocol 2 (65%–8) (p=0.006); conditioning protocol 1 (65%–4) and conditioning protocol 3 (85%–4) (p=0.000); conditioning protocol 2 (65%–8) and conditioning protocol 4 (85%–8) (p=0.002), and between conditioning protocol 3 (85%–4) and conditioning protocol 4 (85%–8) (p=0.002), respectively.

## Discussion

The results of the present study showed that the method of alternating heavy and light resistances had a significant acute effect on power output. Significant increases in mean power output were detected when 65% of 1RM preload stimulus was matched with a 4 min rest interval and 85% of 1RM preload stimulus was matched with an 8 min rest interval. Moreover, there were significant differences in power output as a result of changes in the conditioning protocols. Thus, the selection of the intensity of the preload stimulus (65% 1RM or 85% 1RM) in conjunction with the selection of the intra-complex rest interval (4 min or 8 min) seem to play a vital role in determining the effectiveness of complex training regimens. The hypotheses of the current study were accepted.

Based on the data of the current study, 65%–4 induced a significant mean power output increase. This finding supports those of Baker (2003), who detected power output increases when 6 bench press reps at 65% of 1RM were followed by 5 bench press throws at a fixed resistance, using a 3 min intra-complex rest-interval. The subjects of Baker’s study (2003) were highly trained rugby-league players with at least one year of experience in complex training regimens. In the current study, participants with no prior experience in complex training were included, which suggests that complex training is also applicable for athletic populations with no background in complex training.

In the current study, 65%–8 induced a non-significant mean power output decrease between pre- and post-conditioning. The power output augmentation following the conditioning contractile activity is highly reliant on the degree to which the contractile mechanisms are fatigued and the degree to which they are potentiated (Brandenburg, 2005). Although an 8 min rest interval would have allowed for full phosphocreatine resynthesis and fatigue dissipation (Bevan et al., 2009), it is likely that the decay rate of the potentiating effect of the submaximal 65% 1RM preload stimulus was substantially increased by that time. Four minutes seemed to be the adequate time frame to elicit power output increases when 65% 1RM preload stimuli were applied as conditioning contractions.

The third conditioning protocol (85%–4) induced a significant mean power output decrease which is in contrast to the results reported by other researchers; for instance, Matthews et al. (2009) demonstrated that the velocity of an explosive basketball push-pass was significantly increased following 5 bench presses at 85% of 1RM with a 4 min rest interval, while Evans et al. (2000) reported significant improvements in medicine ball putt distance after the application of 5 bench press reps at 87.5% 1RM with 3 min rest between the strength and power exercise. Moreover, Marcovic et al. (2008) documented significant improvements in maximal throwing speed after the application of 2 sets of 3 bench presses at a 92.5% 1RM load with 3 min rest between the strength and power exercise. However, similar to the findings of the current study, Brandenburg (2005) failed to demonstrate a significant power output increase when the preload stimulus of 87.5% 1RM preceded three concentric bench press throws at 45% 1RM with a 4 min intra-complex rest interval, while Hrysomallis and Kidgell (2001) did not report any significant improvements in the performance of explosive push-ups following a heavy resistance 87.5% 1RM bench press set.

Studies that confirm the findings of the present research have utilized either the bench press throw exercise or biomechanically similar plyometric exercises (i.e. push-ups) in order to measure power output. In contrast, studies that have displayed contradictory results have mainly utilized medicine ball exercises to assess power output generation. It seems that more insightful conclusions could have been reached if the mode of the power exercise was standardized across the research studies testing the 85%–4 conditioning protocol. A proposed elucidation accounting for the lack of improvement in upper-body power output generation following the third conditioning protocol (85%–4) is the presence of fatigue (Brandenburg, 2005). It seems that the intra-complex rest interval of 4 min after the heavy load set at 85% of 1RM was not adequate to allow for a net potentiated state. However, when the applied rest interval increased to 8 min, significant acute increases in mean power output were observed.

The majority of researchers have utilized rest intervals of around 4 min in order to allow a neuromuscular fatigue recovery process to occur after the heavy pre-load stimulus (Docherty et al., 2004). However, the fourth conditioning protocol (85%–8) of the current study induced significant mean power output increases. Bevan et al. (2009) demonstrated that bench press power output, could be significantly improved following a heavy resistance-training pre-load stimulus, provided that an adequate rest interval of 8 min was given between the strength and the power exercise (Bevan et al., 2009) confirming Nevill et al. (1997). Moreover, Fereira et al. (2012) observed significantly increased explosive bench press performance (6 reps at 50% 1RM) at an interval of 7 min when 100% 1RM was used as the conditioning activity. However, despite maximal increases in peak power output, individual determination of the optimal rest interval may be essential when trying to take advantage of any potential effect of PAP on performance (Docherty and Hodgson, 2007).

The current study detected significant differences between the four distinct conditioning protocols. Up to our knowledge, this is the first study that attempted to investigate the effectiveness of complex training regimens by manipulating both the preload stimulus and rest interval. These significant differences demonstrated that the manipulation of complex training variables (preload stimulus and rest interval) can have an impact on the subsequent power output production and can impinge on the effectiveness of a complex training regimen. In contrast to the results of the current study, Brandenburg (2005) failed to detect significant differences in the potentiation ratio produced by four different experimental protocols (87.5% 1RM with 4 min rest, 66% 1RM with 4 min rest, 44%1RM with 4 min rest, and a control group). The absence of significant differences between the distinct conditioning protocols may be attributed primarily to the fact that the fatigue produced exceeded the potentiation generated, and secondarily to the controlled velocity of the lifts, which may have in turn resulted in less effective neural activation (Brandenburg, 2005). It must be acknowledged that Brandenburg (2005) utilized a constant rest-interval of 4 minutes, whereas the current study manipulated the variable of the rest interval (4 and 8 minutes).

A limitation of the current study was the lack of electromyographic recordings and as a result the potential mechanism for the increase in power output production in the corresponding experimental conditions can only be speculated. It has been suggested that individuals need to possess a certain level of strength/training in order to yield the potential benefits of PAP (Chiu et al., 2003; Hodgson et al., 2005). Although not all athletes in the current study were significantly strong, they were all conditioned to high-level training. Thus, the differences in power output observed in the current study may be partially explained by the training status of the participants and by the high proportion of fast-twitch muscles fibers that they possibly possess (Terzis et al., 2009).

Another likely potential mechanism that has been proposed and has possibly accounted for the positive changes in power output is the stiffness of the musculo-tendinous unit and particularly the series elastic component (Baker, 2003). This mechanism is highly dependent on the resistance to be overcome (Wilson et al., 1991); 65% of 1RM load enables higher lifting speeds than the 85% of 1RM and can attune the neural output to a higher speed thereby increasing the chances of neural adaptations in the subsequent faster power exercise (Baker, 2003). The results of this study cannot provide clear evidence to support the theoretical basis of the muscoltendinous stiffness, however, it can be speculated that the resistance of 65% of 1RM is bound to result in a favorable temporal increase in series elastic component stiffness while the resistance of 85% of 1RM will possibly result in a stiffer than optimal series elastic component given the lighter resistance to be overcome in the power exercise (Wilson et al., 1991). Nevertheless, the heavy load resistance should probably be at least twice the lighter load resistance in order to allow for the requisite stimulatory effect on the neuromuscular system (Baker, 2003).

An increase in power output can emerge when sets of heavy load strength exercises are alternated with sets of lighter load power exercises. However, even when a conditioning protocol of that nature may not yield power output increases, methods of training that combine and alternate between a heavy load strength exercise and a lighter load power exercise (plyometric) in a single training session can at least offer a time-efficient training approach that may not undermine the training quality and effectiveness of the plyometric exercises (Brandenburg, 2005). In the current study, it was demonstrated that matching the heavy preload stimulus with the appropriate intra-complex rest interval is of utmost importance in order to elicit an increase in power production. Thus, for optimal results, 85% of 1RM preload stimuli may be matched with 8 min rest intervals and 65% of 1RM with 4 min rest intervals. In the applied training setting it may be advisable to even individually determine the rest interval to optimize the stretch shortening cycle and achieve maximum results (Comyns et al., 2006). Strength and conditioning coaches should experiment with a range of loads and rest intervals when performing complex training in order to find the best possible complex training regimen for a given athlete (Matthews et al., 2009).

## Figures and Tables

**Table 1:** Physical characteristics of participants at baseline

Variables	Mean ± SD
Body mass (kg)	76.9±6.2
Body height (cm)	181.0±7.6
Age (yr)	26.1±3.4
1 Repetition Maximum (kg)	83.9±8.4

**Table 2:** Bench Press Power before and after Conditioning Protocols

	PRE-COND (Mean ± SD) (W)	POST-COND (Mean ± SD) (W)	(POST-PRE) COND (Mean ± SD) (W)
65%–4	530.3 ± 65.9	556.8 ± 79.4 ^**^	26.44 ± 13.78
65%–8	538.0 ± 61.8	530.8 ± 56.2	−7.22 ± 9.13 ^†,§^
85%–4	541.7 ± 55.1	537.1 ± 57.2 ^*^	−4.56 ± 4.95 ^†,§^
85%–8	534.2 ± 58.0	554.7 ± 60.63 ^**^	20.9 ± 10.13

PRE-COND= Pre-conditioning protocol mean power and standard deviation, POST-COND= Post-conditioning protocol mean power and standard deviation, POST—PRE COND= Mean power change

* p=0.001,

** p=0.025,

† Significant differences between 65%–4 and 65%–8, (p =0.006), 85%–4, (p = 0.000),

§ Significant differences between 85%–8 and 65%–8, 85%–4 (p =0.002)
